# Erasing and Refining Discriminative Features for CNV Subtype Classification in OCT Images

**DOI:** 10.3390/jimaging12090405

**Published:** 2026-08-27

**Authors:** Jiayi Zhang, Qingbo Wang, Jiqiang Liu, Aixi Qu

**Affiliations:** 1 Beijing Key Laboratory of Security and Privacy in Intelligent Transportation, Beijing Jiaotong University, Beijing 100044, China; 25115270@bjtu.edu.cn; 2 Shandong Institute for Product Quality Inspection, Jinan 250102, China; shandamla@163.com; 3 School of Computer and Artificial Intelligence, Shandong Jianzhu University, Jinan 250101, China

**Keywords:** choroidal neovascularization, fine-grained classification, salient features, subtle discriminative features, attention-oriented feature fusion

## Abstract

Choroidal neovascularization (CNV) subtype classification from optical coherence tomography (OCT) images is clinically important because treatment response and disease prognosis vary across subtypes. However, automated classification remains challenging owing to subtle inter-class differences and considerable imaging noise. We propose an erasing–refining discriminative feature network (ERDF-Net) that mitigates noisy dominant activations and reveals fine-grained structural cues. The model perturbs salient regions to facilitate subtle feature learning, restores clean salient information, and fuses both representations via channel–spatial attention to form a coherent and discriminative embedding of CNV morphology. Experiments on a clinical OCT dataset show that ERDF-Net consistently surpasses state-of-the-art fine-grained and erasing-based methods across multiple metrics. Ablation and visualization analyses further confirm the benefit and interpretability of controlled salient suppression and refined feature fusion. ERDF-Net provides an effective and reliable solution for fine-grained medical image classification.

## 1. Introduction

Choroidal neovascularization (CNV) is characterized by the pathological growth of choroidal vessels through Bruch membrane into the subretinal or subretinal pigment epithelium space [1]. This process may lead to subretinal or intraretinal fluid accumulation, hemorrhage, exudation, and fibrosis, ultimately causing visual impairment or blindness if not effectively treated [2,3]. Optical coherence tomography (OCT) has become the primary modality for CNV evaluation owing to its non-invasive imaging mechanism, rapid acquisition, and ability to provide high resolution visualization of retinal microstructures [4,5,6]. It supports both qualitative and quantitative assessments of CNV [7,8] and is widely used for routine monitoring in macular disease management [9].

In clinical practice, CNV is classified into Type-1, Type-2, and mixed type according to the anatomic location of the neovascular complex relative to the RPE [10]. Although intravitreal anti-vascular endothelial growth factor (anti-VEGF) therapy is the standard of care [11], treatment regimens vary among CNV types. For example, Type-1 lesions typically require three initial monthly injections, whereas Type-2 lesions often need fewer treatments during induction [12]. CNV typing further influences decision-making and prognosis in laser photocoagulation and photodynamic therapy [10,13]. These clinical dependencies highlight the essential role of precise CNV typing.

**Figure 1:**
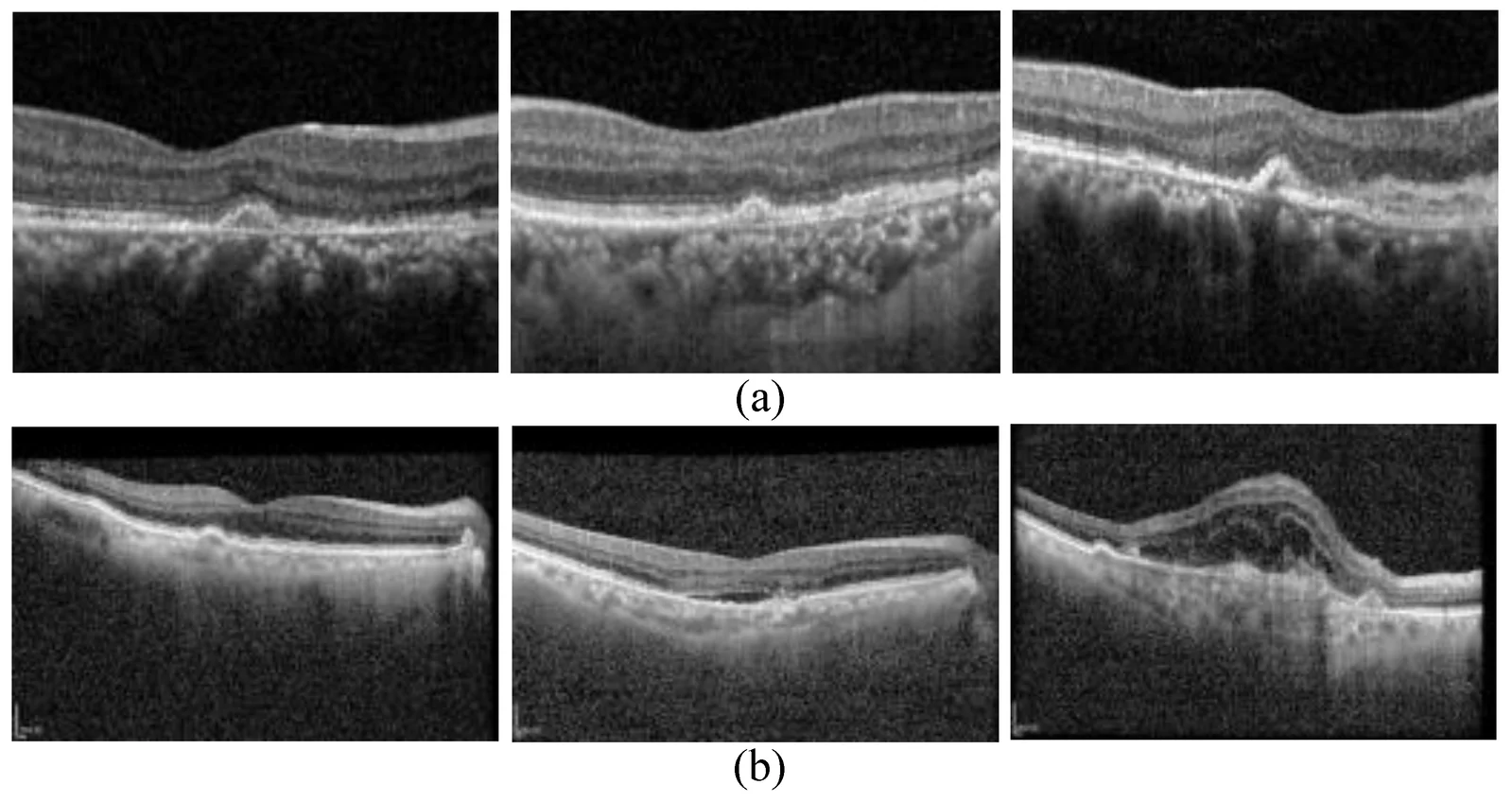
Illustration of several OCT images from CNV dataset. (**a**) The images from left to right, respectively, belong to Type 1, Type 2, and Mixed type. (**b**) Large amounts of noise are present in the CNV OCT images.

However, fine-grained CNV typing remains highly challenging. OCT manifestations of different CNV subtypes vary only in subtle structural cues, resulting in extremely small inter-class variation. As illustrated in Figure 1a, the structural differences are visually minute even for experienced specialists. Additionally, speckle noise and heterogeneous artifacts commonly present in OCT images (Figure 1b) further obscure discriminative details. Existing fine-grained classification methods have explored region suppression and input perturbation to encourage the discovery of local discriminative cues. For example, the destruction–construction method in [14] perturbs input regions and reconstructs local patches to guide attention toward discriminative areas, while the progressive erase network (PEN) [15] employs multi-grid region erasing to reduce reliance on dominant patterns. Although these approaches encourage the model to explore less salient regions, their perturbation mechanisms mainly operate at the input or region level rather than explicitly reshaping the underlying intermediate feature representations. As a result, the network may still relearn dominant salient features instead of discovering truly overlooked subtle structures, while remaining vulnerable to noise in OCT images.

To address these limitations, we propose an Erasing and Refining Discriminative Feature Network (ERDF-Net) that explicitly rebalances the learning of salient and subtle cues for CNV typing. ERDF-Net comprises two collaborative modules. The finer feature mined (FIFM) module suppresses salient features learned during early training, compelling the model to mine previously neglected local subtle discriminative features, and subsequently reconstructs denoising salient features through a dedicated refinement mechanism. The attention-oriented feature fusion (AOFU) module then integrates the denoised salient features with local subtle discriminative features and employs channel–spatial attention to enhance their complementary information. This hierarchical erasing–refining–fusing process yields a more complete and noise-resilient representation, effectively mitigating inter-class similarity and strengthening CNV subtype discrimination.

The main contributions of this work can be summarised as follows:

(1) We introduce an erasing and refining framework that systematically mines discriminative cues, providing a solution to the imbalance between salient and subtle features in fine-grained medical image classification.
(2) We design the FIFM module to suppress early salient dominance, mine fine-grained local structures, and reconstruct reliable global information.
(3) We develop the AOFU module to adaptively fuse and enhance complementary subtle-salient features, substantially improving recognition of visually similar CNV subtypes.

## 2. Related Work

### 2.1. Choroidal Neovascularization Typing

Early attempts at automated CNV analysis relied on unsupervised learning. A k-means-based clustering framework was proposed in [16], where 13 predefined parameters were used to group CNV and age-related macular degeneration samples. However, this approach provided only coarse classification and lacked the capability to capture subtype-specific characteristics. Subsequent deep learning models have demonstrated improved performance. For example, a six-block convolutional network was introduced in [10] to classify diabetic macular edema, CNV, and drusen. Yet, these networks were not designed to distinguish subtle structural differences among CNV subtypes and thus do not achieve precise subtype classification.

More recent work has explored detail-sensitive feature learning. A progressive training network incorporating a detail feature learning module was introduced in [17], enabling the extraction of local nuances through hierarchical information fusion. Another study [18] developed a feature enhancement architecture combining class-specific feature extraction and attention-based region selection to jointly learn discriminative and diverse subtype features under the constraints of multiple loss functions. Although these methods improve representation learning, they primarily emphasize salient regions and still struggle to mine the fine-grained discriminative cues essential for separating visually similar CNV subtypes.

### 2.2. Fine-Grained Image Classification

Fine-grained image classification aims to distinguish subtypes within a broader class set [19,20,21]. Previous studies have shown that subtle local cues are often more informative than global structures for capturing inter-class nuances [14,22]. With the rapid development of deep learning, fine-grained methods have evolved from hand-crafted, multi-stage pipelines [23] to end-to-end convolutional neural network (CNN)-based frameworks [24,25]. A representative direction is the ’destruction–construction’ paradigm, which enhances sensitivity to local variations by deliberately weakening dominant patterns. For example, the method in [15] disrupts input images using a region confusion mechanism and subsequently reconstructs local patches to guide attention toward discriminative areas. The progressive erase network (PEN) [14] further strengthens this paradigm by employing multi-grid region erasing to emphasize local discriminative cues, while pixel-level erasing disrupts global structures to reduce reliance on salient but coarse patterns. More recently, a siamese self-supervised framework [26] incorporates attention-guided semantic mining and multi-view alignment to learn view-invariant fine-grained representations.

Despite these advances, most existing approaches operate solely at the image level, relying on augmentation-based erasing or perturbation. Such pixel-level disruptions do not directly regulate latent feature representations, making it difficult to suppress dominant feature activations once they re-emerge through network training. Consequently, models tend to reattend to the same salient structures, limiting their ability to capture fine-grained, class-specific patterns. Moreover, existing methods lack mechanisms to denoise salient representations, which is essential in OCT-based clinical tasks where speckle noise and heterogeneous artifacts severely degrade discriminative features.

## 3. Method

### 3.1. Overall Framework

**Figure 2:**
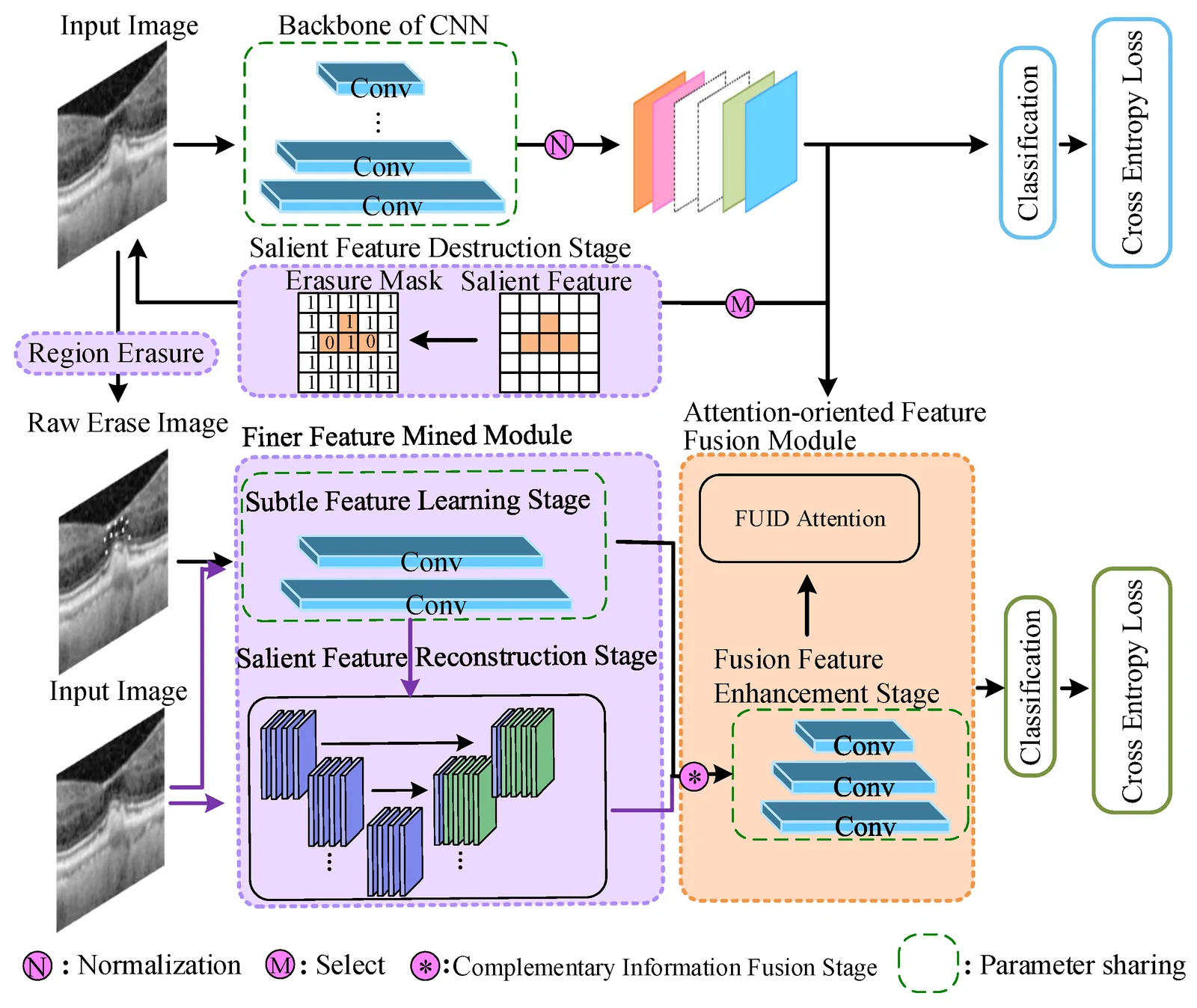
The framework of the proposed ERDF-Net: two-stage training ((**Top**): initial training stage; (**Bottom**): secondary training stage).

To address the challenge that inter-class differences in CNV mainly appear as subtle local variations, we develop ERDF-Net to enhance the extraction and integration of discriminative fine-grained cues from OCT images (Figure 2). The framework contains a backbone feature extractor followed by two complementary modules, FIFM and AOFU. These modules work together to suppress dominant salient responses, mine subtle structural information, and refine fused representations to achieve reliable CNV typing.

Given an input OCT image $I_{o}$, a backbone network such as ResNet-50 [27] obtains an initial feature map $F\in R^{C\times W\times H}$, where *C*, *W* and *H* indicate channels, width, and height, respectively. The feature map *F* is first processed by the FIFM module. FIFM comprises three stages: a salient feature destruction stage that perturbs dominant activations in the feature space, a subtle feature learning stage that encourages the network to discover previously suppressed local cues, and a salient feature reconstruction stage that denoises and restores reliable global structures. The outputs of FIFM are two complementary streams: mined subtle features and reconstructed salient features.

The AOFU module receives these two streams and performs complementary information fusion followed by attention-guided refinement. Specifically, AOFU aggregates subtle and salient feature maps, applies convolutional refinement, and uses channel and spatial attention to enhance their complementary discriminative content. The resulting fused representation is forwarded to a classifier $C_{class}$ composed of two fully connected layers and a softmax layer to produce the predicted probability vector $y^{pre}$. The network is trained using the cross entropy loss between $y^{pre}$ and the groundtruth label *y*.

(1) $$L_{\mathrm{ce}}=-y\log\left(y^{pre}\right).$$

### 3.2. Finer Feature Mined Module

The FIFM module is designed to uncover subtle discriminative cues that are often overlooked when the backbone relies excessively on dominant salient activations. It consists of three stages: salient feature destruction, subtle feature learning, and salient feature reconstruction.

#### 3.2.1. Salient Feature Destruction Stage

Our proposed salient feature destruction stage aims to suppress dominant salient features that are insufficient for discriminating visually similar CNV subtypes, thereby facilitating subsequent learning of local subtle discriminative cues.

After training the backbone network using the cross-entropy loss, we obtain the feature map *F*. Each feature map is normalized to produce $F^{\prime }$ as

(2) $$F^{\prime }=\frac{F_{i}-F_{\min}}{F_{\max}-F_{\min}},\;i=1,\ldots ,W\times H,$$

where $F_{\max}$ and $F_{\min}$ represent its maximum and minimum values.

Based on the normalized feature responses, salient feature regions are identified by applying a threshold *T*, which is empirically determined via performance evaluation experiments (see Section 4.6). The salient region mask $f_{\mathrm{area}}$ is defined as:

(3) $$f_{\mathrm{area}}=Mask\left(F^{\prime }>T\right),$$

where Mask(·) denotes a binary labeling function.

To suppress these salient regions at the image level, the generated salient mask is mapped back to the input space. A random erase mask $f^{\prime }$ is constructed such that only the salient regions are randomly assigned values of 0 or 1, while all non-salient regions are preserved:

(4) $$f^{\prime }=\left\{\begin{array}{cc} random(0,1), & f^{\prime }\in f_{\mathrm{area}},\\ 1, & f^{\prime }\notin f_{\mathrm{area}}. \end{array}\right.$$

Finally, the erase mask $f^{\prime }$ is applied to the raw input image $F_{r}$ through element-wise multiplication to generate the erased image $F_{re}$:

(5) $$F_{re}=F_{r}⊙f^{\prime },$$

**Figure 3:**
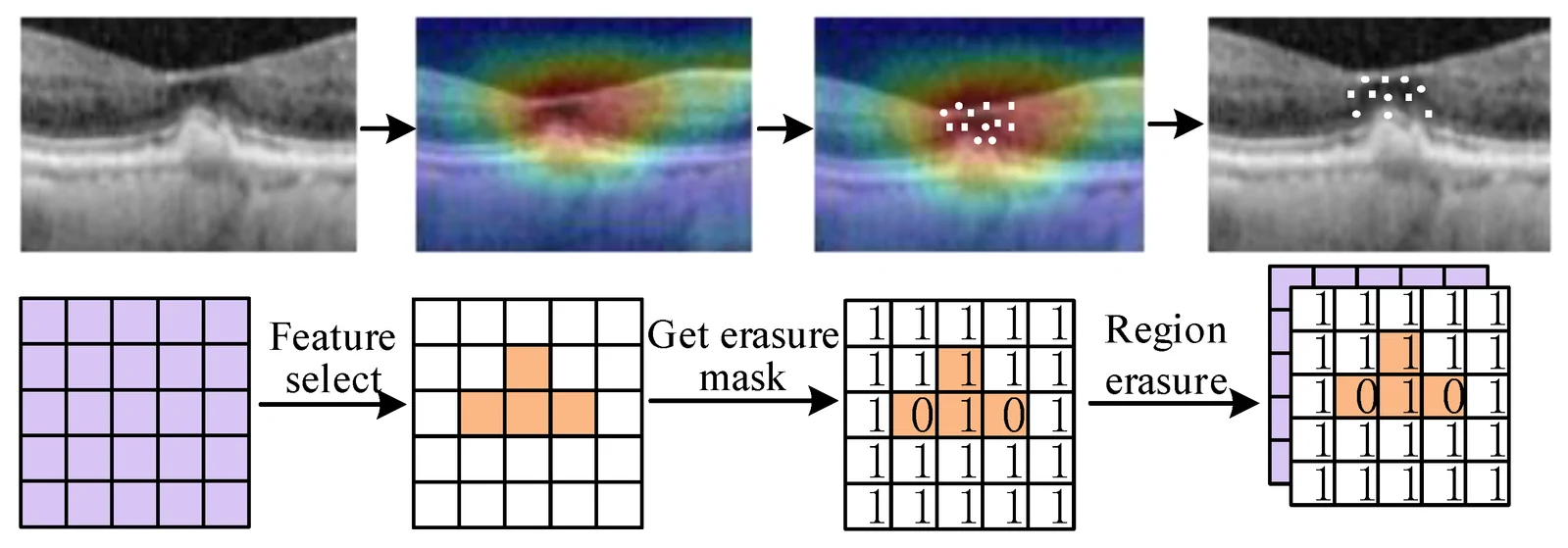
Process of salient feature destruction in two different forms. The first line is from the image perspective, and the second line is from the perspective of image matrix.

As illustrated in Figure 3, the salient feature destruction process is presented from two complementary perspectives: the image-level view (top row), which shows salient region localization derived from backbone features and random erasing applied to the raw OCT image, and the matrix-level view (bottom row), which provides an abstract matrix representation of the same saliency-guided erasing operation.

This operation selectively suppresses dominant salient regions in the original input while preserving non-salient areas, thereby providing appropriate inputs for subsequent subtle feature learning.

#### 3.2.2. Subtle Feature Learning Stage

To extract subtle discriminative features overlooked in the initial training, the backbone network is retrained using parameters initialized from the first training stage. Under the constraint of the cross-entropy loss, the first two stages of the backbone are optimized to relearn fine-grained representations from shallow layers. Both the erased feature maps $F_{\mathrm{re}}$ and the original input images $I_{o}$ are used as inputs to enhance sensitivity to local subtle structures [28]. The resulting feature maps are computed as

(6) $$f_{v}=B^{F}\left(I\right),$$

where $f_{v}$ includes subtle feature maps $f_{s}$ and shallow feature maps $f_{sa}$, $I\in \{F_{\mathrm{re}},I_{o}\}$, and $B^{F}\left(\cdot \right)$ denotes the first two stages of the backbone network.

#### 3.2.3. Salient Feature Reconstruction Stage

**Figure 4:**
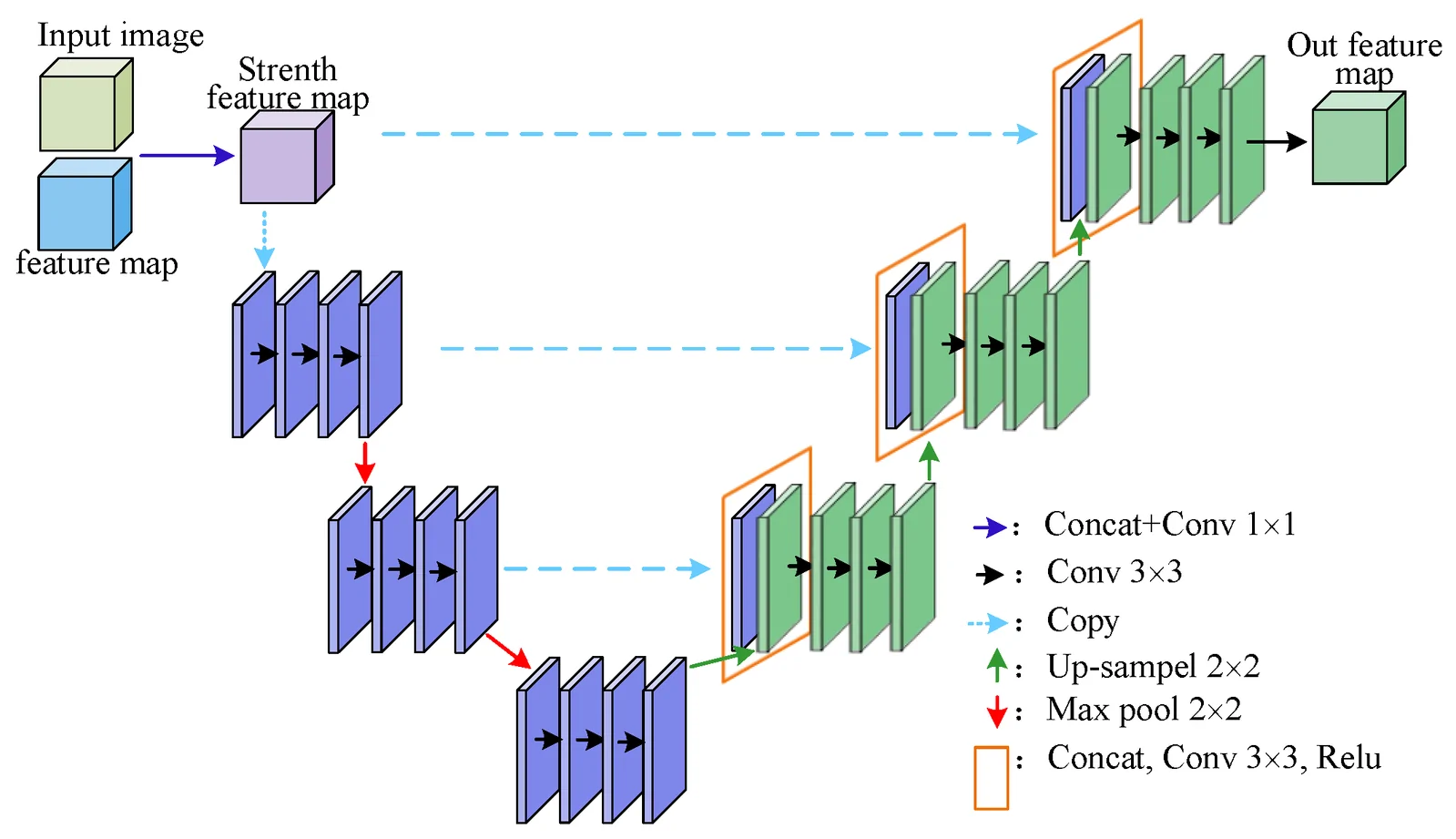
The process of the salient feature reconstruction stage.

As illustrated in Figure 1, CNV OCT images often exhibit complex lesion structures and substantial noise. Denoised salient representations are therefore critical for robust classification. Motivated by prior work showing that successive down-sampling and up-sampling operations facilitate denoising [29], this stage employs three cascaded down-sampling and up-sampling blocks, with bilinear interpolation used for up-sampling, as shown in Figure 4. Skip connections are introduced to preserve both shallow visual information and high-level semantic features.

To further restore fine image details, the original input *I* and shallow feature maps $f_{sa}$ are fused through concatenation followed by a 1×1 convolution. The input image *I* is first processed by two convolution–BatchNorm–ReLU blocks:

(7) $$f_{i+1}=\mathrm{ReLU}\left(\mathrm{BN}\left(\mathrm{Conv}\left(f_{i}\right)\right)\right),\;i\in \left\{0,1\right\},$$

where $f_{0}=I$. This reconstruction stage outputs denoised and semantically enriched salient feature maps.

### 3.3. Attention-Oriented Feature Fusion Module

Given the subtle inter-class differences and pervasive noise in CNV OCT images, the attention-oriented feature fusion (AOFU) module is designed to learn robust and discriminative representations by integrating denoised salient features with local subtle features. The AOFU module consists of a complementary information fusion stage and a fusion feature enhancement stage.

#### 3.3.1. Complementary Information Fusion Stage

**Figure 5:**
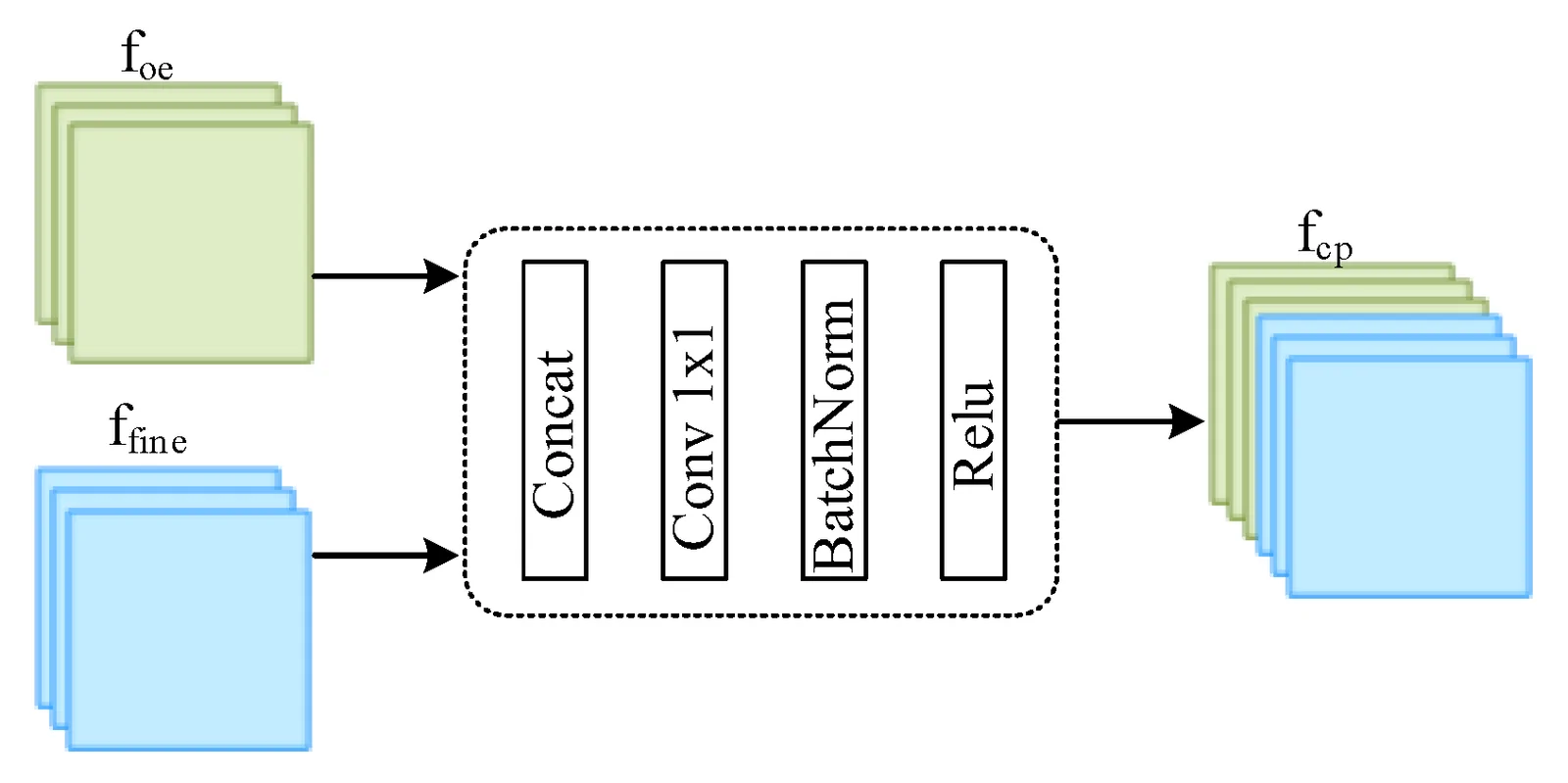
Process of complementary information fusion.

Accurate CNV typing requires both global structural context and fine-grained local details. Relying solely on denoised salient features or subtle features is insufficient, as each lacks complementary information. Therefore, this stage fuses the denoised salient features $f_{dn}$ and subtle discriminative features $f_{s}$ to form a unified representation. As illustrated in Figure 5, the complementary feature map $f_{cp}$ is obtained by concatenation followed by convolution, BatchNorm, and ReLU operations:

(8) $$f_{cp}=\mathrm{ReLU}\left(\mathrm{BN}\left(\mathrm{Conv}\left(\mathrm{concat}\left(f_{s},f_{dn}\right)\right)\right)\right).$$

#### 3.3.2. Fusion Feature Learning Stage

The fused feature map $f_{cp}$ is fed into the last three stages of the backbone network to learn deeper semantic representations. The parameters from the corresponding stages of the pretrained backbone are used for initialization to stabilize training and enhance feature learning.

#### 3.3.3. Fusion Feature In-Depth Learning Attention

**Figure 6:**
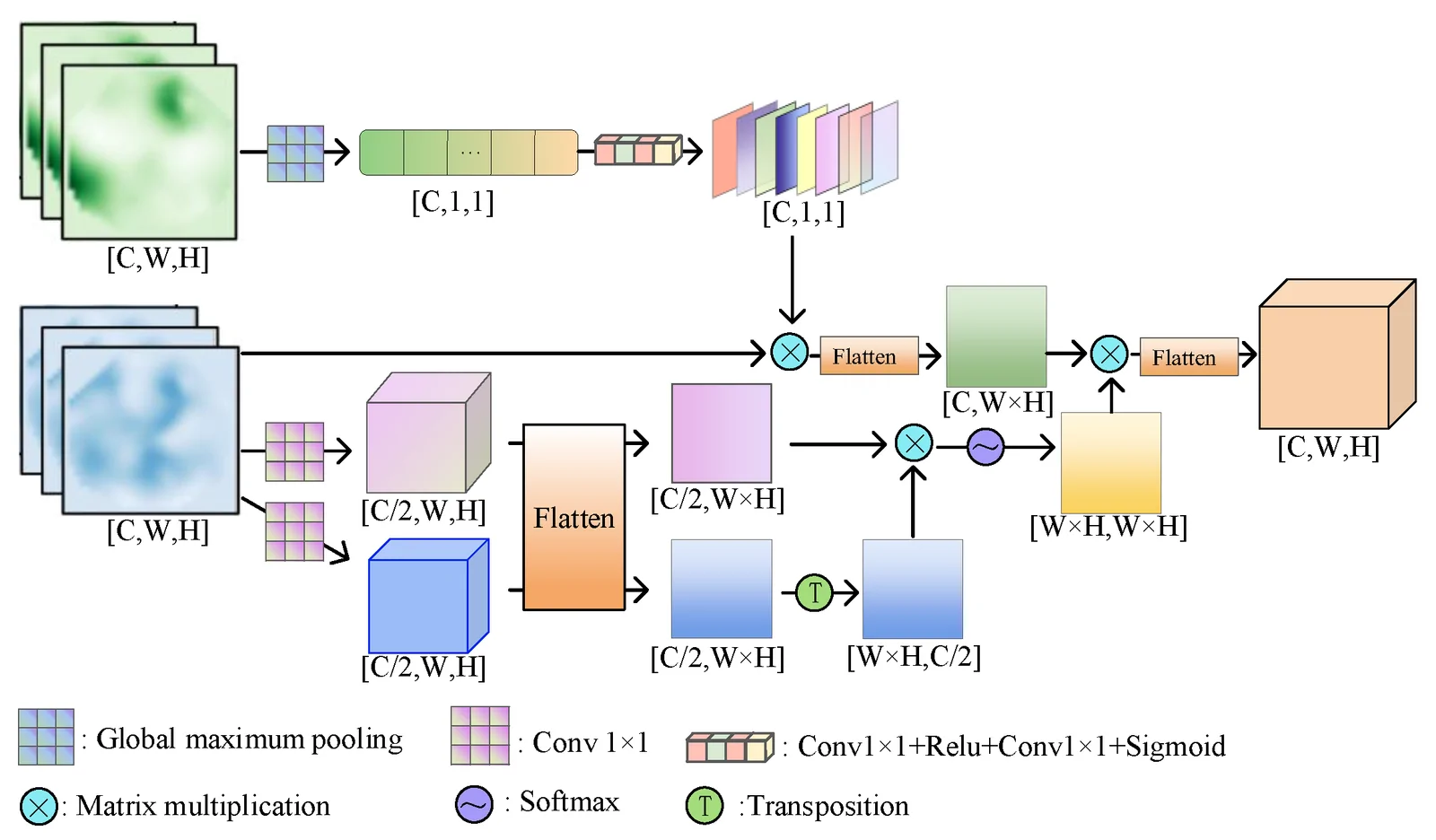
An illustration of fusion feature in-depth learning attention (FUID).

To further enhance the fused features, channel and spatial attention mechanisms [30] are employed, as shown in Figure 6. Channel attention emphasizes informative channels and is defined as

(9) $$F^{c}=M_{c}\left(f\right)⊙f,$$

where $M_{c}\left(\cdot \right)$ denotes channel attention computed via global average pooling, global max pooling, convolution, and element-wise addition. Based on the channel-refined features, spatial attention is applied to highlight discriminative spatial regions:

(10) $$F^{s}=M_{s}\left(F^{c}\right)⊙F^{c},$$

where $M_{s}\left(\cdot \right)$ is derived from max pooling, average pooling, convolution, and concatenation operations. This attention-guided refinement yields the final enhanced feature representation for CNV classification.

### 3.4. Training and Inference

ERDF-Net is trained in two steps. In the first step, the network is optimized using raw OCT images to obtain a stable backbone initialization. In the second step, the backbone is reinitialized with the first step parameters, and the network is retrained using both the raw image and its erased feature representation. The same backbone and classifier parameters are shared across the two steps, and cross entropy loss is used throughout. During inference, ERDF-Net uses the parameters learned in the second training stage. The network operates on raw OCT images without performing salient-feature erasing. FIFM and AOFU process the clean feature representation to produce the final classification score.

## 4. Experiments

### 4.1. Dataset

We evaluated ERDF-Net using a clinical OCT dataset provided with the assistance of Qianfoshan Hospital in Shandong Province. OCT images were acquired using a Heidelberg Spectralis OCT system with standardized clinical acquisition protocols. The dataset consists of 380 patients, including 20 CNV Type-1 patients, 150 CNV Type-2 patients, and 210 CNV mixed-type patients. This study was a retrospective analysis based on previously collected clinical OCT images. Patients were included if they were clinically diagnosed with CNV and had OCT images of sufficient quality for subtype classification. Cases with severe image artifacts or insufficient clinical information were excluded.

All OCT images were fully anonymized and de-identified before analysis, and no personally identifiable information was available to the researchers. The ground truth labels were established through repeated confirmation by several experienced ophthalmologists to ensure the reliability of the annotations. The dataset used in our experiments is identical to that described in [17].

### 4.2. Experiment Setting

We evaluated ERDF-Net using four standard metrics: accuracy (ACC), area under the ROC curve (AUC), sensitivity (SEN), and specificity (SPE). ACC measures the proportion of correctly classified samples, AUC reflects overall discrimination ability, SEN denotes the proportion of correctly identified positives, and SPE quantifies the correctly rejected negatives. The metric definitions are as follows:

(11) $$ACC=\frac{T_{p}}{T_{t}},$$

(12) $$SEN=\frac{1}{3}\sum_{n=1}^{3}\theta \left(\frac{T_{n}^{+}}{T^{+}}\right),$$

(13) $$SPE=\frac{1}{3}\sum_{n=1}^{3}\theta \left(\frac{T_{n}^{-}}{T^{-}}\right),$$

where $T_{t}$ represents the amounts of test samples, $T_{p}$ indicates the correct amounts of classifications, and $\theta \left(n\right)$ means the amounts of *n* type of CNV image as a true sample, where n∈{1,2,or mixed}, and the amounts of the other two types as false samples, such as the type 1 as a true sample and the type 2 and the mixed type as false samples. $T^{+}$ indicates the amounts of true samples, and $T_{n}^{+}$ means the amounts of correctly predicted true samples. $T^{-}$ denotes the amounts of false samples, and $T_{n}^{-}$ indicates the amounts of correctly predicted false samples.

**Figure 7:**
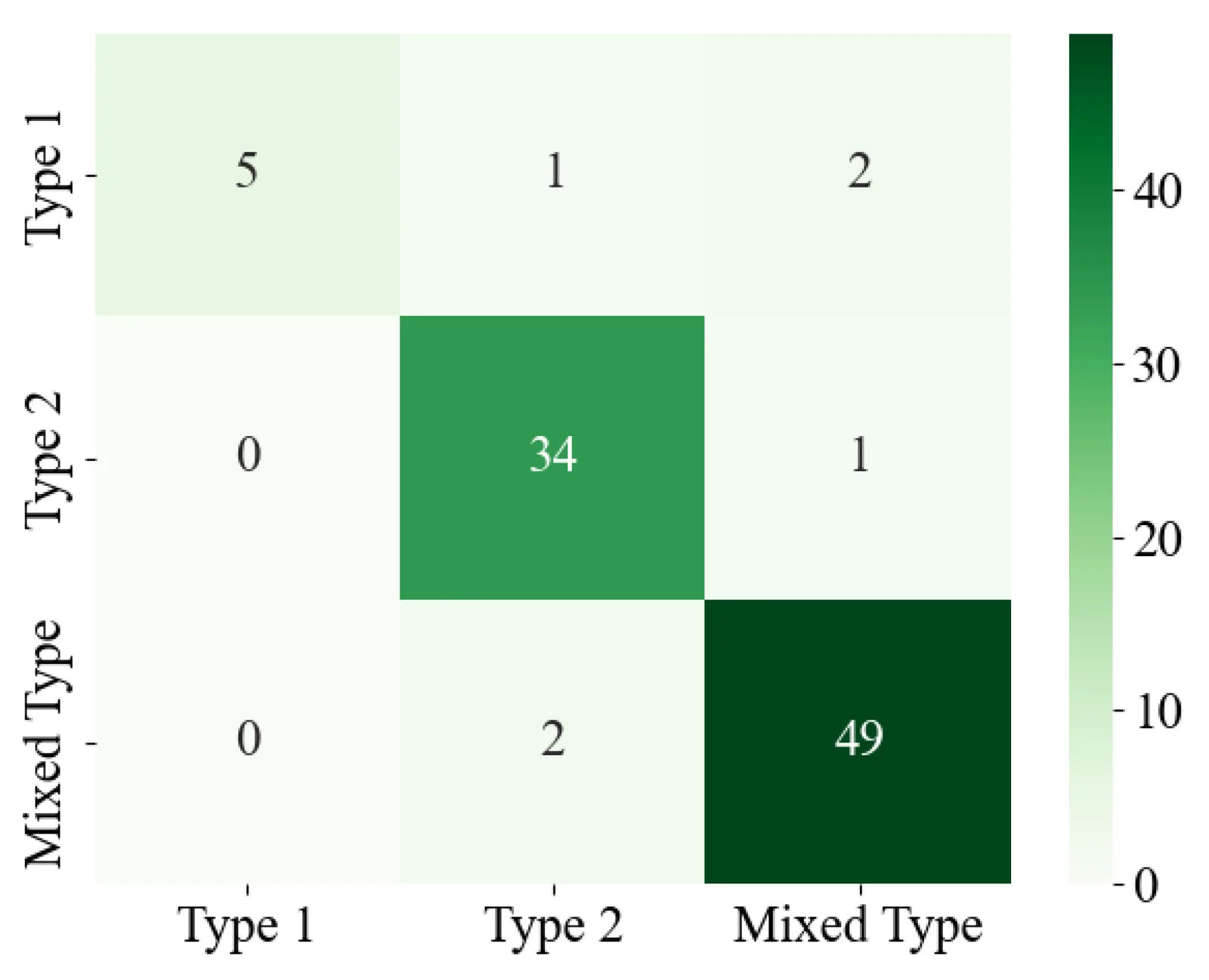
The confusion matrix on ACC indicator of ERDF-Net for different CNV types.

Four-fold cross-validation was performed on the entire dataset with patient-level partitioning. In each fold, three subsets were used for training and the remaining subset was used for testing, ensuring that images from the same patient did not appear in both the training and test sets. The final performance was reported as the average across the four folds. To mitigate class imbalance, the training partitions in each fold were augmented by random translation, cropping, and flipping, increasing each class to 2000 images and yielding 6000 images in total. The confusion matrix in Figure 7 presents the results from one test fold, in which ERDF-Net achieved accuracies of 100%, 91.89%, and 94.23% for Type-1, Type-2, and mixed-type CNV, respectively. Considering the limited number of independent Type-1 patients, we further evaluated Type-1 classification across all four folds. ERDF-Net achieved an average test accuracy of 95% for Type-1 CNV across the four folds, providing a more stable assessment of its classification performance.

All images were resized to 224×224 pixels. ResNet-50 was used as the backbone feature extractor. The model was optimized using stochastic gradient descent. The learning rate of pretrained convolutional layers was set to $1\times 10^{-3}$, and that of the remaining layers was initialized to $1\times 10^{-3}$ and decayed following the cosine annealing schedule [23]. The training was performed with a batch size of 16, a weight decay of $5\times 10^{-4}$, and an early-stopping strategy.

### 4.3. Comparison with State-of-the-Art Methods

Table 1 reports the performance of ERDF-Net compared with several representative fine-grained classification approaches, including DFL-CNN [24], LDE-net [17], TASN [31], MC-Loss [25], as well as FE-Net [18]. ERDF-Net achieves the highest average ACC, AUC, SEN, and SPE across the dataset, reaching 93.75%, 88.17%, 92.13%, and 91.84%, respectively. Compared with the strongest baseline, FE-Net [17], ERDF-Net improves ACC by 1.42% and SEN by 2.03%, while also achieving higher AUC and SPE. These results demonstrate that the proposed erasing–refining strategy effectively strengthens the model’s ability to distinguish visually similar CNV subtypes.

To further evaluate performance, Table 2 compares ERDF-Net with commonly used image-level erasing strategies, including Cutout [32], Random Erasing [33], Hide-and-Seek [34], and GridMask [35]. ERDF-Net achieves the highest mean ACC, SEN, and SPE among these methods, whereas Random Erasing obtains a slightly higher mean AUC than ERDF-Net (88.81% vs. 88.17%). Conventional image-level erasing methods mainly suppress input regions to encourage the exploration of overlooked features, but the removed information is not explicitly recovered, which may result in incomplete representations. In contrast, ERDF-Net adopts a perturbation-restoration mechanism, where FIFM suppresses dominant responses to promote subtle feature mining and reconstructs reliable salient information to preserve complementary structural cues. This design provides a more complete representation than single erasing strategies for noisy OCT images.

Considering that only four-fold cross-validation was adopted in this study, the reliability of statistical inference is limited. Therefore, we further calculated the 95% confidence intervals (CIs) for the ACC comparison with FE-Net and the AUC comparison with Random Erasing, which exhibited comparable performance. For ACC, ERDF-Net achieves a 95% CI of [90.82, 96.68]%, compared with [90.15, 94.51]% for FE-Net. For AUC, the corresponding 95% CIs of Random Erasing and ERDF-Net are [84.89, 92.73]% and [85.00, 91.34]%, respectively. The overlap of the corresponding CIs reflects the variability of four-fold cross-validation results. Nevertheless, ERDF-Net maintains the best mean performance on ACC, SEN, and SPE, supporting its effectiveness in learning discriminative representations for CNV subtype classification.

### 4.4. Ablation Study

#### 4.4.1. Backbone Ablation Study

**Figure 8:**
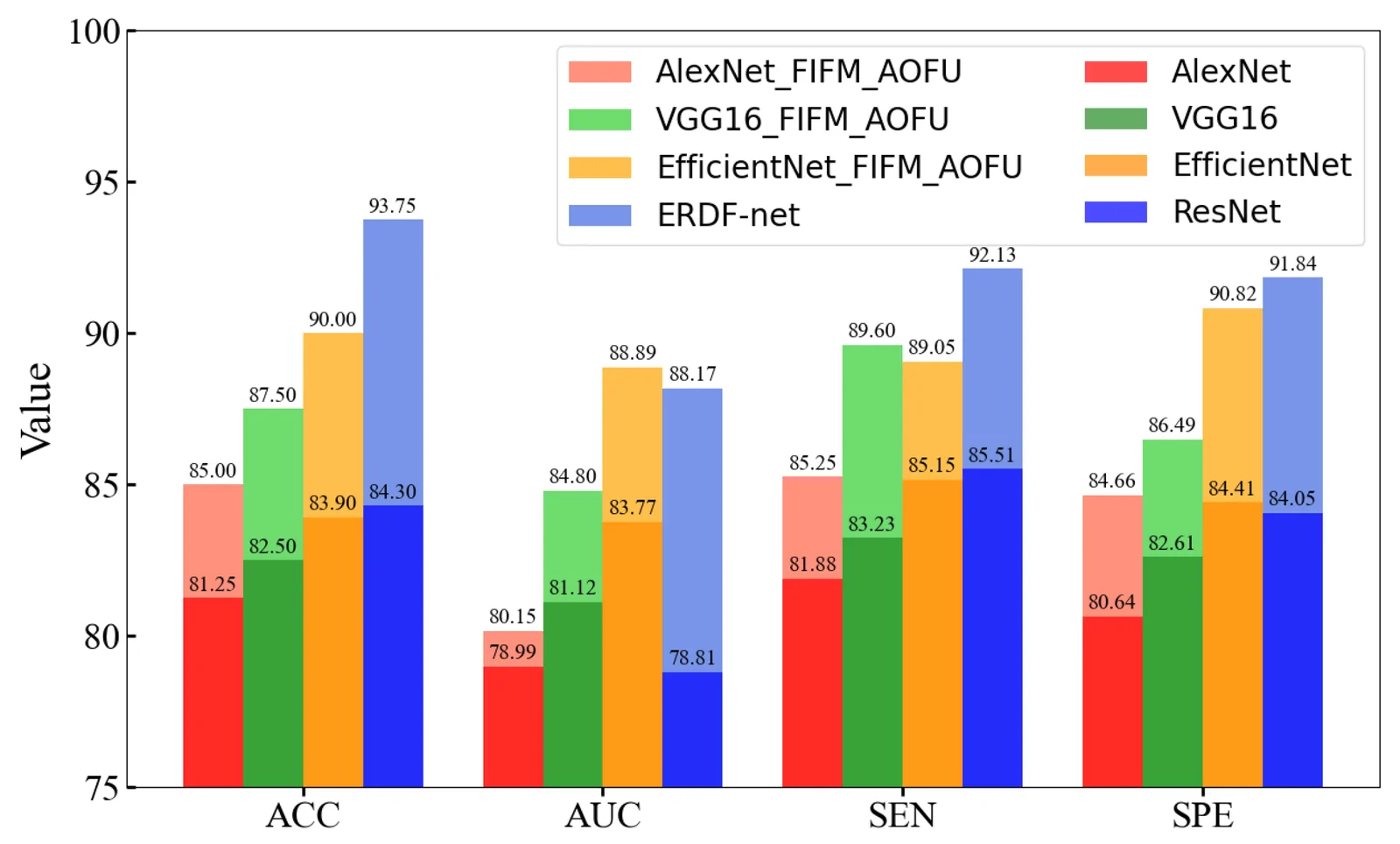
Performance comparison of different backbone networks with and without the proposed FIFM and AOFU modules.

To evaluate the applicability of FIFM and AOFU across different architectures, we integrated both modules into four commonly used backbone networks (AlexNet [36], VGG [37], EfficientNet [38] and ResNet [27]). The results in Figure 8 show that, for all backbones, adding FIFM and AOFU consistently improves ACC, AUC, SEN, and SPE compared with the corresponding baseline models. The gains are more pronounced in shallower networks such as AlexNet and VGG16, indicating that the proposed modules effectively compensate for their limited ability to capture fine-grained cues. Stronger backbones like EfficientNet and ResNet50 also benefit from the integration, demonstrating that FIFM and AOFU provide complementary fine-detail extraction and robust feature fusion that generalize well across different feature extractors.

#### 4.4.2. Component Ablation

To assess the contribution of each module, ablation experiments were conducted, and the results are summarized in Table 3. The ResNet-50 backbone serves as the baseline. Adding the AOFU module leads to the most substantial individual improvement, increasing ACC by more than 6% along with consistent gains in AUC, SEN, and SPE. Incorporating the FIFM module alone also improves performance, though with a smaller ACC increase of about 2%. When both FIFM and AOFU are integrated, ERDF-Net achieves the highest overall performance, with ACC improving by nearly 10% over the baseline. These results confirm that FIFM and AOFU provide complementary contributions. FIFM improves classification by encouraging the extraction of subtle discriminative cues from suppressed salient responses, while AOFU further enhances the complementary information through attention-guided fusion. Their combination achieves the best performance, demonstrating that both fine-grained feature mining and adaptive feature integration are essential for CNV subtype classification.

#### 4.4.3. Attention Ablation

We further examined the design of the attention mechanism used in AOFU by replacing the original channel–spatial attention module [30] with several widely used alternatives, including Self-attention [39], Non-local blocks [40], SE [41], Triple-Net [42], DANet [43], CA-Net [44], and MSCA [45]. As shown in Table 4, all replacements result in performance degradation. The channel–spatial attention used in ERDF-Net sequentially models channel relationships and spatial saliency, which is particularly effective for refining the fused subtle-salient representation produced by FIFM. In contrast, the alternative mechanisms emphasize either global dependencies or a single feature dimension, leading to less effective enhancement of the fused features. ERDF-Net thus maintains the highest ACC and AUC, confirming that the adopted attention scheme is well aligned with the design goals of AOFF and is essential for achieving optimal performance.

#### 4.4.4. Two-Stage Training Ablation

To evaluate the effectiveness of the proposed two-stage training strategy, we compared it with one-stage training under identical network architecture and experimental settings. As shown in Table 5, the proposed two-stage strategy achieves better performance than direct one-stage training across all evaluation metrics. This improvement indicates that the first-stage training provides a stable salient-feature reference, enabling more effective feature destruction and subtle discriminative feature mining in the second stage. By progressively refining feature representations rather than directly optimizing with erased inputs, the two-stage strategy helps preserve reliable structural information while enhancing the discovery of complementary cues for CNV subtype classification.

### 4.5. Visualization Analysis

**Figure 9:**
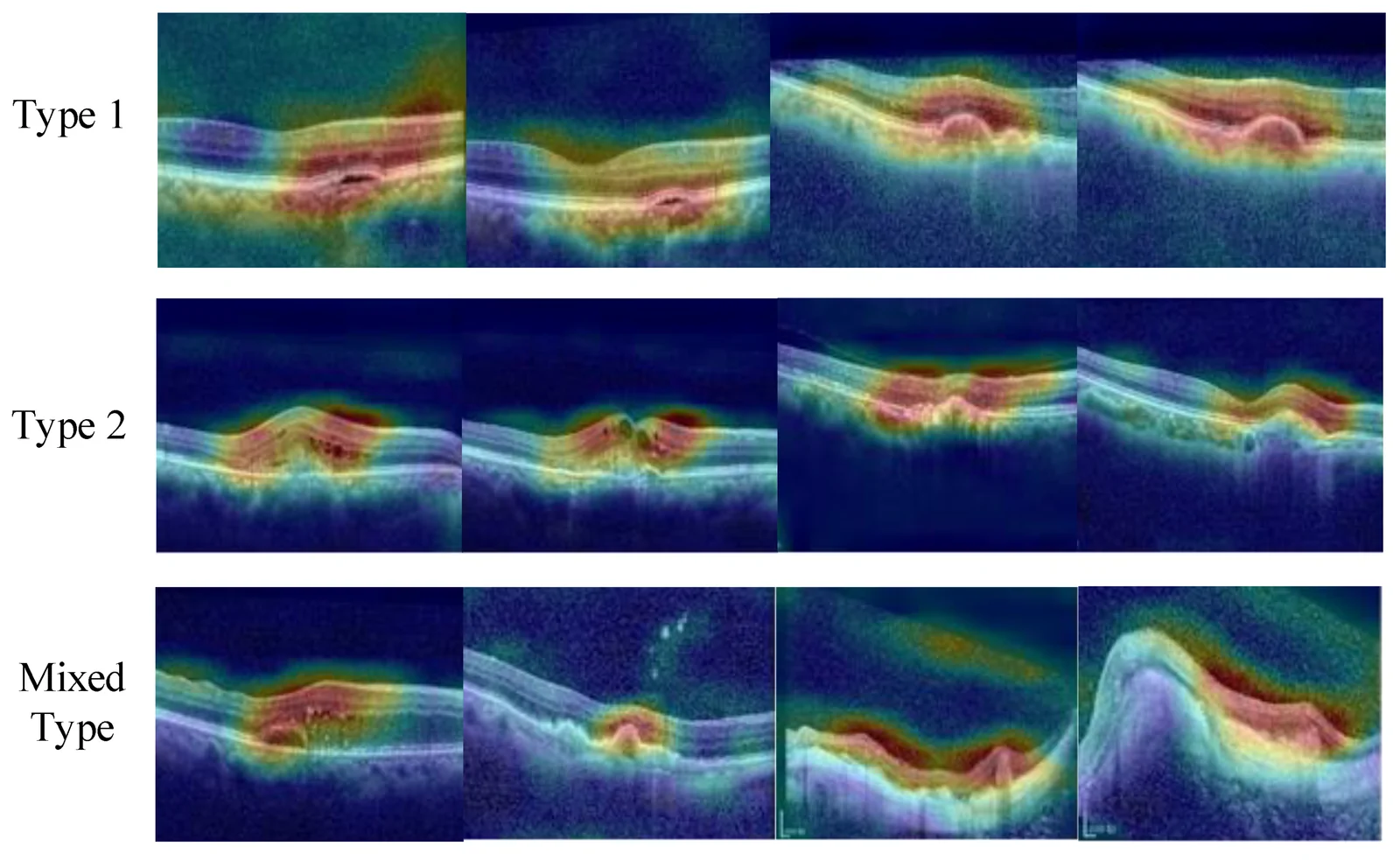
Visualizations of ERDF-Net’s activation maps for representative samples from the three CNV subtypes.

Figure 9 presents the activation maps of ERDF-Net for representative samples from the three CNV subtypes. The highlighted regions are mainly distributed around clinically meaningful retinal structures, including lesion-associated regions near the retinal pigment epithelium (RPE) interface and abnormal retinal layers, which are important for CNV subtype differentiation. These activation patterns indicate that ERDF-Net focuses on disease-related morphological changes rather than irrelevant background regions.

Across different CNV subtypes, the activated regions show consistency with their characteristic morphological patterns. For Type-1 CNV, ERDF-Net mainly highlights the elevated regions beneath the RPE, corresponding to the typical location of type 1 neovascular lesions. For Type-2 CNV, the highlighted regions are concentrated around subretinal structural abnormalities associated with neovascular lesions. Mixed-type CNV exhibits broader activation involving multiple retinal layers, reflecting the coexistence of morphological characteristics from both Type-1 and Type-2 CNV. These visualization results suggest that ERDF-Net can effectively capture subtype-related morphological cues from OCT images. The complementary learning of subtle features through FIFM and adaptive feature integration through AOFU enables the model to exploit both local discriminative structures and reliable salient information, thereby improving the accuracy and reliability of CNV subtype classification.

### 4.6. Sensitivity to Erasure Ratio and Threshold T

**Figure 10:**
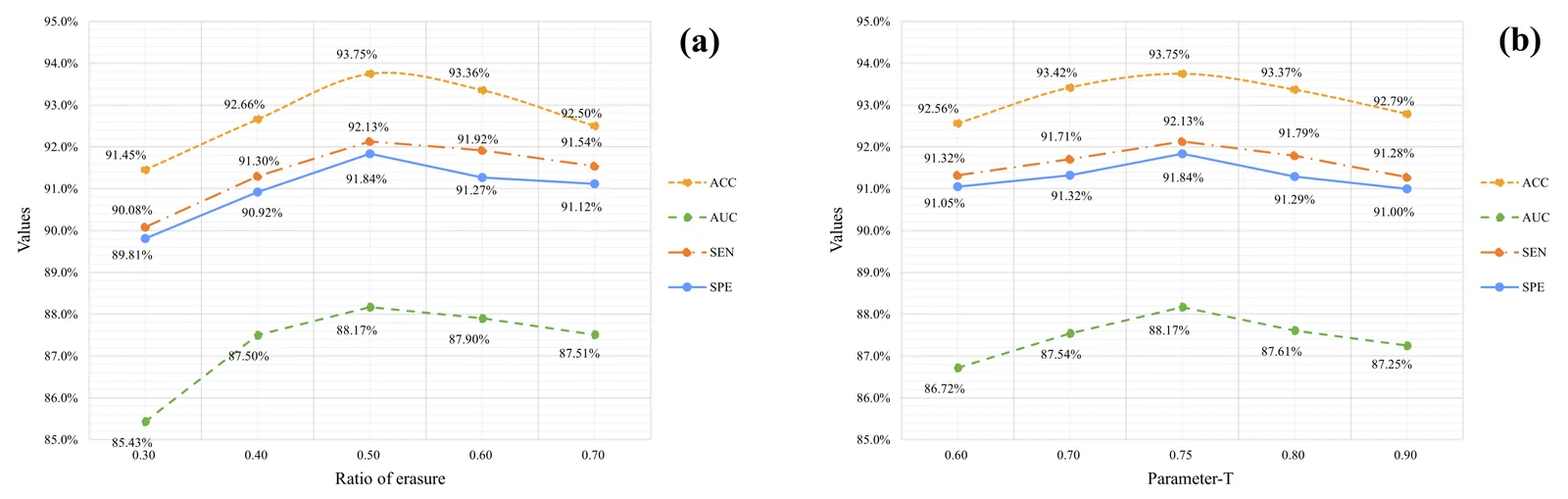
Sensitivity analysis of ERDF-Net with respect to the erasure ratio and threshold *T*. (**a**) Ratio of reasure. (**b**) Parameter-T.

Both the erasure ratio and threshold *T* regulate the salient feature destruction stage, where salient activations ($F^{\prime }>T$) are identified and partially removed to guide the network toward subtle feature mining. As shown in Figure 10a, performance improves steadily as the erasure ratio increases to 0.50, after which all metrics decline, indicating that moderate suppression of salient regions best balances subtle feature excavation and salient feature recoverability. A similar pattern is observed for *T* (Figure 10b): raising the threshold from 0.60 to 0.75 enhances performance by yielding more accurate salient region selection, whereas larger values exclude useful salient structures and reduce accuracy. These results demonstrate that ERDF-Net benefits from controlled salient suppression, with optimal performance achieved near an erasure ratio of 0.50 and T = 0.75.

## 5. Discussion

Accurate CNV subtype classification remains difficult because inter-class differences are subtle and OCT images are heavily affected by noise. Unlike conventional single erasing strategies, ERDF-Net integrates perturbation, restoration, and attention-guided fusion to jointly mine subtle discriminative cues, preserve reliable salient information, and generate more robust representations for noisy OCT images. Specifically, FIFM suppresses dominant responses to encourage subtle feature discovery and reconstructs reliable salient representations, while AOFU adaptively integrates these complementary features through attention-guided fusion. In addition, the clinical OCT dataset contains unavoidable imaging noise and subtype imbalance, particularly the limited number of Type 1 CNV cases. ERDF-Net alleviates these challenges through feature refinement and data augmentation strategies. The feature-level salient suppression and reconstruction mechanism helps reduce the dependence on potentially noisy dominant responses while preserving informative structural cues. Meanwhile, the balanced augmentation strategy during training alleviates the influence of uneven subtype distribution.

The experimental results demonstrate that this design materially strengthens discriminative capacity. Existing fine-grained models often emphasize dominant discriminative regions and thus overlook subtle variations essential for CNV typing, whereas ERDF-Net jointly captures subtle and salient cues and achieves consistently superior performance. Ablation studies confirm that both FIFM and AOFU are integral components, and visualization results show that the model attends to clinically meaningful structures that align with ophthalmologic interpretation.

Despite its strong performance, ERDF-Net has two limitations. First, the salient feature destruction stage requires manually selected parameters such as the erasure ratio and threshold *T*, which increases training complexity during model optimization. However, these parameters do not introduce additional learnable parameters or computational operations during inference, since the deployed model only requires a single forward propagation. Moreover, although FIFM and AOFU introduce additional feature refinement and attention fusion operations, they operate on intermediate feature representations and only introduce limited computational overhead compared with the backbone network. Future work will explore adaptive parameter learning, potentially through meta-learning or uncertainty-driven mechanisms, to further improve training efficiency. Second, the current dataset was collected from a single medical center using a single OCT device. Although ERDF-Net achieved promising performance on this clinical cohort, its robustness to domain shifts across clinical centers and imaging systems requires further validation. Future studies will involve external validation using multi-center datasets and OCT images acquired from different manufacturers to further assess the generalizability and clinical applicability of ERDF-Net.

## 6. Conclusions

This work proposes ERDF-Net, an erasing–refining framework for CNV subtype classification in OCT images. By suppressing dominant activations, mining subtle structures, reconstructing reliable salient cues, and integrating complementary information through attention-guided fusion, the model forms a complete and noise-resilient representation of CNV morphology. ERDF-Net achieves strong performance across multiple evaluation metrics and effectively distinguishes visually similar CNV subtypes. Such automated subtype classification has the potential to assist ophthalmologists in treatment planning, patient management, and prognosis assessment by providing objective and reproducible OCT-based diagnostic information. Future studies involving multi-center datasets and different OCT systems will further evaluate its clinical applicability.

## Figures and Tables

**Table 1:** Comparison between our method and previous state-of-the-art methods on our self-built CNV dataset. Note: Bold values in the table denote the best-performing results.

Model	Backbone	ACC	AUC	SEN	SPE
DFL-CNN [24]	VGG16	88.38 ± 1.07	83.45 ± 1.47	86.58 ± 1.65	87.81 ± 1.64
LDE-net [17]	ResNet50	92.30 ± 1.21	87.11 ± 1.14	91.53 ± 1.52	90.89 ± 1.32
TASN [31]	ResNet50	91.07 ± 1.33	83.06 ± 1.05	91.22 ± 1.39	87.81 ± 1.22
MC-Loss [25]	ResNet50	89.64 ± 1.86	85.94 ± 1.29	88.01 ± 1.57	88.74 ± 1.69
FE-Net [18]	ResNet50	92.33 ± 1.37	87.45 ± 1.32	90.10 ± 1.26	91.25 ± 1.97
ERDF-Net	ResNet50	**93.75 ± 1.84**	**88.17 ± 1.99**	**92.13 ± 2.24**	**91.84 ± 4.41**

**Table 2:** Comparison between our method and previous image-level erase methods. Note: Bold values in the table denote the best-performing results.

Model	ACC	AUC	SEN	SPE
Cutout [32]	92.89 ± 1.62	88.57 ± 2.74	90.12 ± 3.32	90.20 ± 4.23
Hide-and-seek [34]	92.50 ± 1.57	88.21 ± 1.77	90.39 ± 3.42	90.07 ± 4.07
Random Erasing [33]	93.24 ± 1.29	**88.81 ± 2.46**	91.22 ± 4.22	91.80 ± 2.05
GridMask [35]	88.75 ± 2.85	89.39 ± 2.70	83.01 ± 3.84	89.71 ± 3.26
ERDF-net	**93.75 ± 1.84**	88.17 ± 1.99	**92.13 ± 2.24**	**91.84 ± 4.41**

**Table 3:** Performance of components from ERDF-Net on the CNV dataset.

ResNet50	FIFM	AOFU	ACC	AUC	SEN	SPE
✓			84.31 ± 1.33	78.80 ± 1.87	85.51 ± 1.47	84.05 ± 1.06
✓		✓	90.87 ± 1.40	85.91 ± 1.57	87.98 ± 3.85	88.31 ± 3.09
✓	✓		86.23 ± 3.45	80.26 ± 2.13	85.41 ± 1.95	86.19 ± 2.11
✓	✓	✓	93.75 ± 1.84	88.17 ± 1.99	92.13 ± 2.24	91.84 ± 4.41

**Table 4:** Comparison between our method and several widely used attention mechanisms. Note: Bold values in the table denote the best-performing results.

Model	ACC	AUC	SEN	SPE
Self-attention [39]	92.25 ± 2.60	87.31 ± 2.34	91.20 ± 3.66	91.38 ± 5.56
Non-local [40]	93.50 ± 2.13	87.94 ± 4.72	91.94 ± 3.27	91.67 ± 4.17
SE [41]	92.64 ± 2.17	87.71 ± 2.91	91.56 ± 3.84	91.16 ± 3.62
Triple-Net [42]	92.50 ± 1.93	87.91 ± 2.03	91.81 ± 2.20	91.22 ± 4.19
DANet [43]	93.32 ± 3.01	88.00 ± 2.97	91.95 ± 3.62	91.50 ± 5.52
CA-Net [44]	93.25 ± 2.51	87.73 ± 2.90	91.77 ± 3.41	91.07 ± 3.98
MSCA [45]	93.34 ± 1.92	87.82 ± 2.53	91.62 ± 3.64	91.38 ± 3.76
ERDF-Net	**93.75 ± 1.84**	**88.17 ± 1.99**	**92.13 ± 2.24**	**91.84 ± 4.41**

**Table 5:** Ablation study of one-stage and two-stage training strategies. Note: Bold values in the table denote the best-performing results.

Training Strategy	ACC	AUC	SEN	SPE
One-stage	91.97 ± 1.96	87.05 ± 2.08	89.83 ± 2.41	90.21 ± 3.73
Two-stage	**93.75 ± 1.84**	**88.17 ± 1.99**	**92.13 ± 2.24**	**91.84 ± 4.41**

## Data Availability

The datasets presented in this article are not readily available due to technical limitations. Data are available upon reasonable request.
